# Ectopic Expression of a *Fagopyrum esculentum APETALA1* Ortholog only Rescues Sepal Development in Arabidopsis *ap1* Mutant

**DOI:** 10.3390/ijms20082021

**Published:** 2019-04-24

**Authors:** Zhixiong Liu, Yue Fei, Kebing Zhang, Zhengwu Fang

**Affiliations:** 1 College of Horticulture and Gardening, Yangtze University, Jingzhou 434025, China; 201771409@yangtzeu.edu.cn (Y.F.); 201671421@yangtzeu.edu.cn (K.Z.); 2 Institute of Crop Genetics and Breeding, Yangtze University, Jingzhou 434025, China; fangzhengwu88@163.com

**Keywords:** *APETALA1*, *Fagopyrum esculentum*, floral development, flowering, perianth development

## Abstract

*Fagopyrum esculentum* (Polygonaceae: Caryophyllales) exhibits an undifferentiated perianth comprising five showy tepals, which does not completely correspond to the perianth differentiated into typical sepals and petals in most core eudicots. In *Arabidopsis*, the *APETALA1* (*AP1*) gene is involved in specifying sepals and petals development. Here we isolated *AP1* ortholog, *FaesAP1*, and a 2.2kb *FaesAP1* promoter (*pFaesAP1*) from *F. esculentum*. *FaesAP1* expression is mainly detectable in all floral organs and maintains at a high level when tepals elongate rapidly both in pin and thrum flowers. Moreover, the *GUS* reporter gene driven by *pFaesAP1* was activated in flowers where the sepals were intense, but the petals very weak or absent. Additionally, *FaesAP1* ectopic expression in *Arabidopsis ap1-10* mutant rescues sepal development fully, obviously prompting early flowering, but failing to complement petal development. In this study, evidence was provided that the showy tepals in the *F. esculentum* are homologs to core eudicots sepals. Furthermore, these findings show a different perianth identity program in Caryophyllales, suggesting that *AP1* orthologs involved in petal development may evolve independently across different clades of core eudicots. Our results also suggest that *FaesAP1* holds potential for biotechnical engineering to develop early flowering varieties of *F. esculentum.*

## 1. Introduction

The *Arabidopsis APETALA1* (*AP1*) gene is involved in establishing floral meristems, specifying normal perianth whorl (sepals and petals) development [1,2], and suppressing the flower formation in the axils of sepals [3]. *AP1* expression is detected initially throughout the floral meristem during the first stages of floral development, but later restricted to the outer two whorls where the sepal and petal emerging by the *AGAMOUS* (*AG*) gene are mediated negatively [4,5,6]. In some core eudicots, the *AP1* orthologs, such as *ChAP1* from *Cardamine hirsute* and *CDM111* from *Dendrathema grandiflorum*, show functional conservation for determining floral meristem and specifying perianth (sepal and petal) identity [7,8]. However, some *AP1* orthologs from other core eudicots show obviously different functions. For example, the *AP1* ortholog *Bna.AP1.A02* from *Brassica napus* was proved to be involved in plant architecture and yield-related traits [9], and *MPF3* from *Physalis floridana* was required for specifying calyx identity and regulating male fertility [10]. All these data show a very interesting and elaborate scenario for functional evolution of *AP1* orthologs in core eudicots. Previous studies suggested that core eudicots *AP1* orthologs result from gene duplication events in ancestral *euFUL*- or *FUL*-like genes from eudicots or basal angiosperms [11,12]. However, some pre-duplication *FUL*-like genes from basal eudicots were present in broad expression zones and versatile functions [13,14,15]. For example, the *FUL*-like genes from *Papaver somniferum* (Papaveraceae) and *Eschscholzia californica* (Papaveraceae) are expressed in leaves, all developing floral organs and fruits, and regulated flowering, axillary meristem growth, normal sepal and fruit development [13]. In addition, *FUL*-like genes from *Aquilegia coerulea* (Ranunculaceae) showed a similar expression pattern with poppies, but played a key role in regulating leaf and inflorescence development [14]. However, the functional evolution scenario of *AP1* orthologs in the early-diverging core eudicots remains unclear.

**Figure 1:**
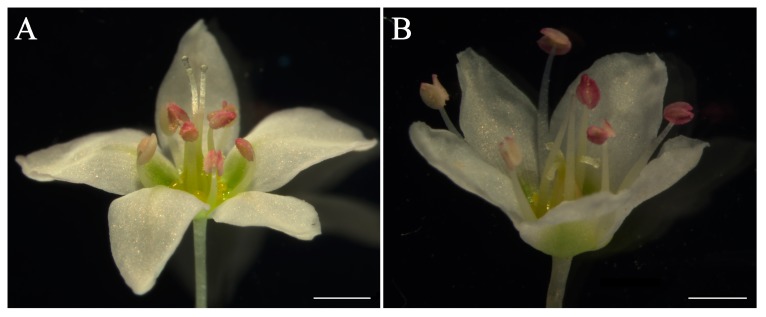
Heterodistylous flowers of *F. esculentum*. (**A**) pin flower with long pistil and short stamens; (**B**) thrum flower with short pistil and long stamens. Scale bar = 1 mm.

*Fagopyrum esculentum* is a member of family Polygonaceae in the Order Caryophyllales, one the early-diverging higher eudicots taxa, producing heterodistylous flowers with showy single-whorled perianths comprising five tepals (Figure 1), representing a considerable difference from most core eudicots flowers [16,17]. Moreover, *F. esculentum* is one of the pseudo-cereal crops with multi-food use and healing benefits, being cultivated in Asia, Europe and North America for a long time [18,19]. Here we isolated an *AP1* orthologous gene, *FaesAP1*, and its promoter from *F. esculentum.* Additionally, the *FaesAP1* promoter activity is evaluated in transgenic *Arabidopsis* using the *β*-glucuronidase (*GUS*) reporter gene, and the flower phenotypes of *FaesAP1* complementing *Arabidopsis ap1-10* mutant are assayed. Our study was conducted in order to understand the functional evolution of *AP1* orthologs in the early-diverging core eudicots, and the perianth identity program in Order Caryophyllales. In addition, the showy tepals of *F. esculentum* homologous to core eudicots sepals or petals are discussed in our study.

## 2. Results

### 2.1. Isolation and Characterization of FaesAP1 from F. esculentum

**Figure 2:**
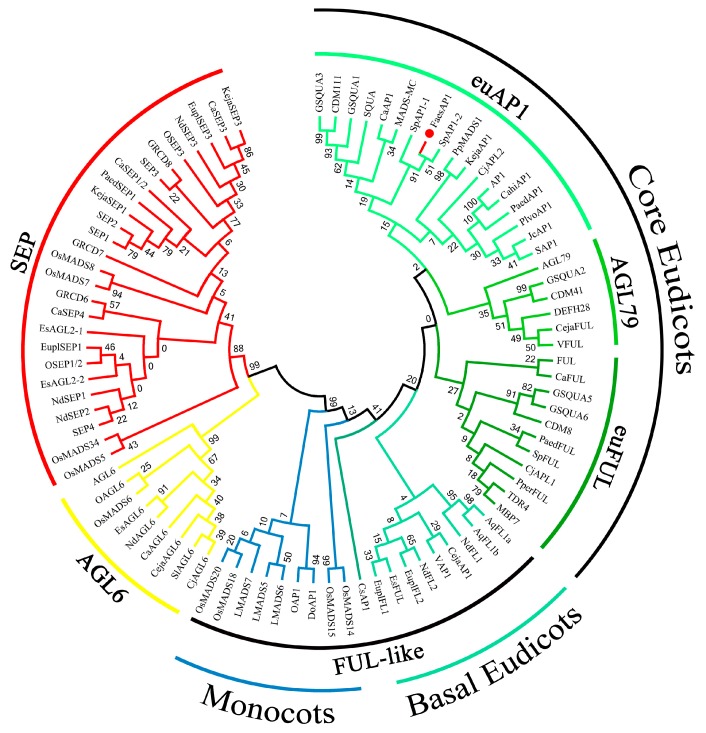
Phylogenetic analysis of FaesAP1 and other AP1/SEP/AGL6 superclade of MADS-box proteins from different clades of angiosperms.

**Figure 3:**
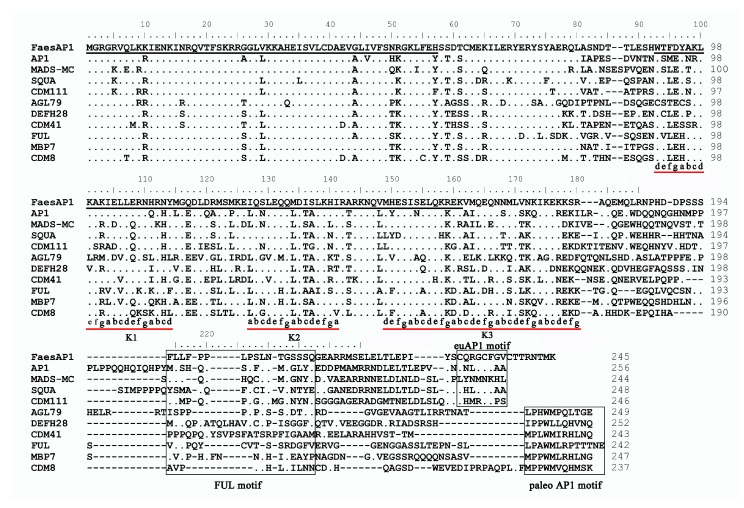
Sequence alignments of FaesAP1 with AP1/FUL-like proteins from model plants. The first underlined region indicates the MADS domain and the second the K domain. The relatively conserved FUL motif, euAP1 motif and paleoAP1 motif located in the various C-terminal region are boxed. Amino acid residues identical to FaesAP1 are indicated as dots. Dashes introduced into the sequence in order to improve the alignment. The k1, k2 and k3 subdomains with (abcdefg)n repeats that usually contain hydrophobic amino acids at positions a and d are underlined [21].

The 1004 bp *FaesAP1* cDNA contains a 738 bp ORF (Open Reading Frame, ORF) encoding 245 amino acids (aa) (Genbank accession number: KM386625.1). Proteins alignment and phylogenetic analysis grouped FaesAP1 into euAP1 lineage (Figure 2). Hence, the gene was designated as *FaesAP1* (*Fagopyrum esculentum APETALA1*). Conceptual translation shows that FaesAP1 protein consists of a 57aa highly conserved MADS-box domain (1–57) at the N-terminal region, a 69 aa moderately conserved K domain (91- 159) in the middle region and a 86 aa variable C-terminal region (160- 245) but with two relatively conserved regions: A FUL motif and an euAP1 motif (Figure 3) [12,20,21]. Moreover, FaesAP1 contains three putative amphipathic α-helices referred to as K1 (91–113), K2 (125–139), and K3 (147–178) subdomains with conserved hydrophobic amino acids at the a and d positions in the (abcdefg)n heptad repeats [21].

### 2.2. Expression Analysis of FaesAP1

**Figure 4:**
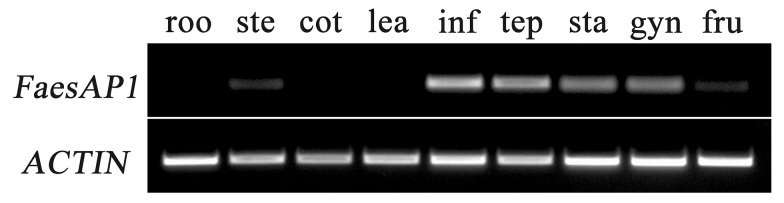
*FaesAP1* expression in the roots (roo), stems (ste), cotyledons (cot), juvenile leaves (lea), inflorescence (inf), tepals (tep), stamens (sta), gynoecia (gyn), and fruits (fru) by sqRT- PCR with ACTIN as the control.

**Figure 5:**
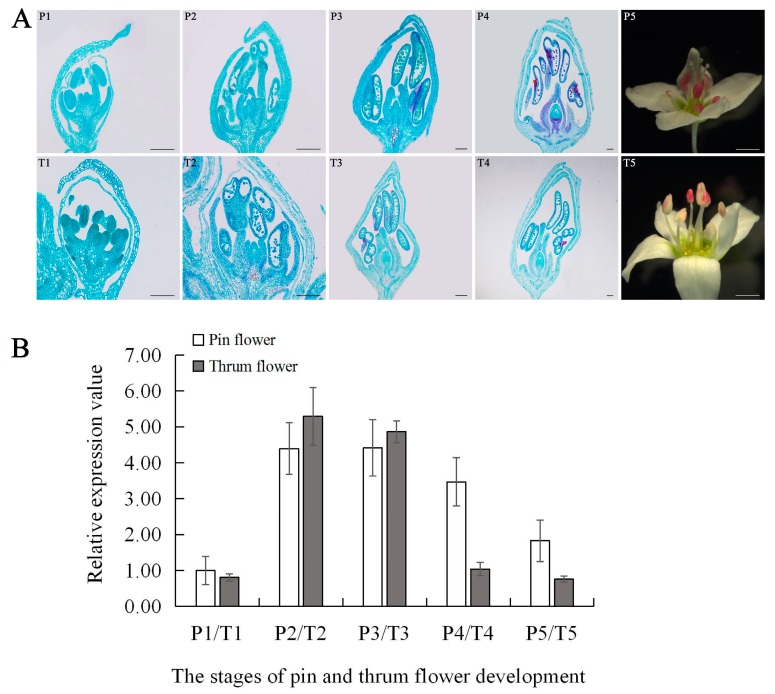
Morphology and *FaesAP1* expression in buckwheat Heterodistylous flowers at different developmental stages. (**A**) Morphology of dimorphic flowers at various development stages; P1–P5: morphological differentiation and development of the pin flower; P1: Tepal primodium, microspore mother cells, and carpel primodium development; P2: Tepal rapid elongating, microspores released from tetrads and megaspore mother cell formation; P3: Tepal enclosing, mononuclear microspore and outer integument emerging; P4: Full maturity flower buds with mature pollen and embryo sac before anthesis; P5: Flower anthesis; T1–T5: Morphological differentiation and development of the thrum flower; T1: Tepal primodium, microspore mother cells and carpel primodium development; T2: Tepal rapid elongating, microspore released from tetrads and megaspore mother cell formation; T3: Tepal enclosing, mononuclear microspore and outer integument emerging; P4: Full maturity flower buds with mature pollen and embryo sac before anthesis; T4: Full maturity flower buds with mature pollen and embryo sac before anthesis; T5: Flower anthesis; (**B**) *FaesAP1* expression at different development stages of pin and thrum flowers was detected by qPCR, respectively. Scale bar: (P1–P4, T1–T4) 200 μm; (P5, T5) 1 mm.

*FaesAP1* transcription was detected mainly in inflorescence, tepals, stamens and gynoecia, but was absent in roots, cotyledons and juvenile leaves. Low transcription was also detected in stems and fruits (Figure 4), which suggests that the *FaesAP1* function may be involved in flower development. The *FaesAP1* expression was detectable at the pin flower P1 stage when tepals began to develop (Figure 5A,B). Moreover, *FaesAP1* expression increased and reached a high level at Pin flower P2 stage during the tepal’s rapid elongation and microspores released from tetrads, and was maintained at a high level at P3 stage when tepals enclosing stamens and gynoecia, before decreasing gradually as the flower buds achieved maturity (P4) and anthesis (P5) (Figure 5A,B). In addition, *FaesAP1* showed a similar expression in thrum flowers (Figure 5A,B). *FaesAP1* expression accumulated in thrum flowers at the T1 stage when tepal primordia formed, and achieved a high level at thrum flower T2 stage when tepal rapid elongating, maintaining at a high level till the tepal enclosing stamens and gynoecia in T3 stage, before dropping as the thrum flower buds reached maturity (T4) and flowering (T5). However, *FaesAP1* expression at late development stages in Pin flower (P4, P5) was higher than the late development stages of thrum flower (T4, T5) (Figure 5A,B).

### 2.3. Isolation and Characterization of the FaesAP1 Promoter

A 2.2 kb *FaesAP1* promoter fragment (−1959/+240) was isolated from *F. esculentum*, and the putative transcription start site and cis-acting regulatory elements of *FaesAP1* promoter (*pFaesAP1*) were shown in Figure S1 (Supplementary Figure S1). *pFaesAP1* contains an important CArG-box (−490/−481) for DNA-binding by MADS-box proteins [22]. Moreover, *pFaesAP1* also contains seven pollen-specific elements POLLEN2LELAT52-boxes [23]. All above data suggest that *FaesAP1* is involving in floral development. In addition, there are several MYCCONSENSUSAT-, MYB1AT- and MYCATERD1-boxes lying in *FaesAP1* promoter region, which suggests that the gene expression may induced by dehydration-/cold-stress [24,25]. In addition, the mesophyll-specific elements CACTFTPPCA1-boxes, root hair-specific elements RHERPATEXPA7-boxes, and secondary-xylem-specific elements XYLAT-boxes have been also found in the *pFaesAP1* region, which suggested that *FaesAP1* expression may extend to vegetable tissues [26,27,28].

**Figure 6:**
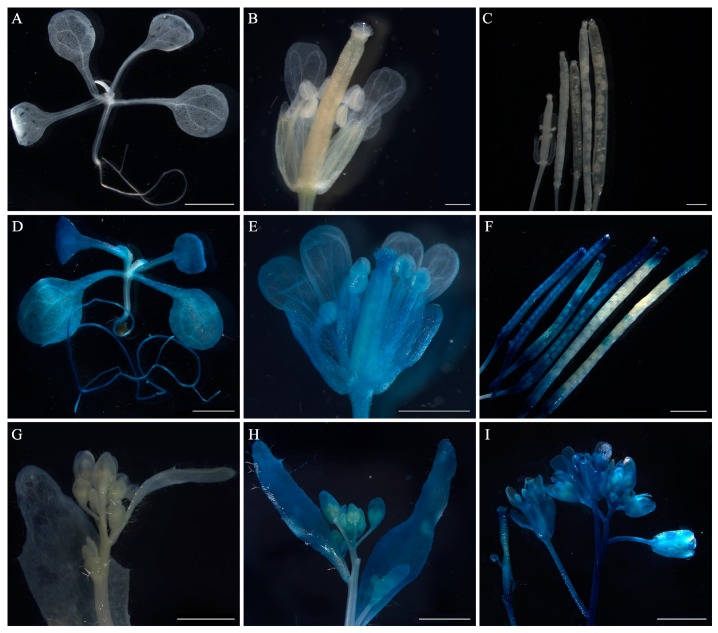
Histochemical GUS staining of *pFaesAP1::GUS* transgenic Arabidopsis. (**A**) Wild-type Arabidopsis seedling; (**B**) wild-type Arabidopsis flower; (**C**) wild-type Arabidopsis fruits at various development stages; (**D**) seedling of *pFaesAP1::GUS* transgenic Arabidopsis; (**E**) flower of *pFaesAP1::GUS* transgenic Arabidopsis; (**F**) *pFaesAP1::GUS* transgenic Arabidopsis fruits at different development stages; (**G**) inflorescence of wild-type Arabidopsis; (**H**) *pFaesAP1::GUS* transgenic Arabidopsis inflorescence before anthesis; (**I**) *pFaesAP1::GUS* transgenic Arabidopsis inflorescence after anthesis. Scale Bars: (**B**) 500 μm; (**C**,**E**) 1 mm; (**A**,**D**,**F**,**G**,**H**,**I**) 2 mm.

A GUS reporter gene driven by the *FaesAP1* promoter (*pFaesAP1*) was activated in the root, cotyledon, and juvenile leaves of transgenic seedling (Figure 6D). Moreover, GUS staining was observed in the flowers where sepals staining was intense, but almost absent in petals (Figure 6E,H), beginning to accumulate in the old rachis, stamen, and gynoecia after anthesis (Figure 6E,I). In addition, the GUS activity was later detectable in the seed capsule of young siliques and terminal part of the old siliques of the transgenic *Arabidopsis* (Figure 6F).

### 2.4. Ectopic Expression of FaesAP1 in Arabidopsis ap1-10 Mutant

**Figure 7:**
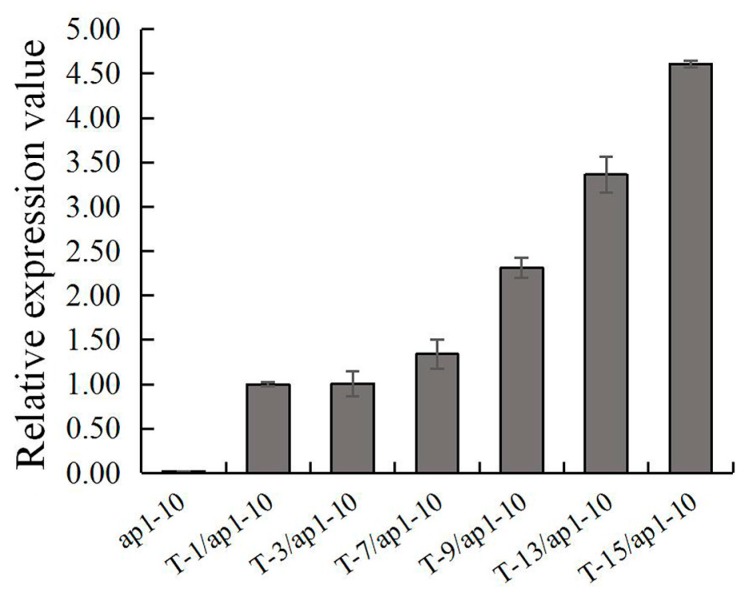
Expression of *FaesAP1* in transgenic Arabidopsis *ap1-10* mutant confirmed by qPCR. *ap1-10*: Arabidopsis *ap1-10* mutant; (*T-1/ap1-10, T-3/ap1-10*) T-1 and T-3 independent lines of 35S:: *FaesAP1* transgenic Arabidopsis ap1-10 mutant with no phenotype complementation; (*T-7/ap1-10, T-9/ap1-10*) T-7 and T-9 independent lines of *35S:: FaesAP1* transgenic Arabidopsis ap1-10 mutant with medium complementation phenotypes only showing early flowering; (*T-13/ap1-10, T-15/ap1-10*) T-13 and T-15 independent lines *35S:: FaesAP1* transgenic Arabidopsis ap1-10 mutants showing early flowering and strong complementation phenotypes.

To explore *FaesAP1* roles regulating floral development, *35S:: FaesAP1* constructs have been transformed into homozygous *Arabidopsis ap1-10* mutants to create complementation lines. Using PCR and qPCR detection (Figure 7), 15 independent *35S::FaesAP1* lines of homozygous transformants under *ap1-10* mutant background were obtained. Phenotypes of transgenic lines were analyzed to evaluate whether *FaesAP1* could substitute for the endogenous *AP1* gene in *Arabidopsis ap1-10* mutant in specifying perianth development.

**Figure 8:**
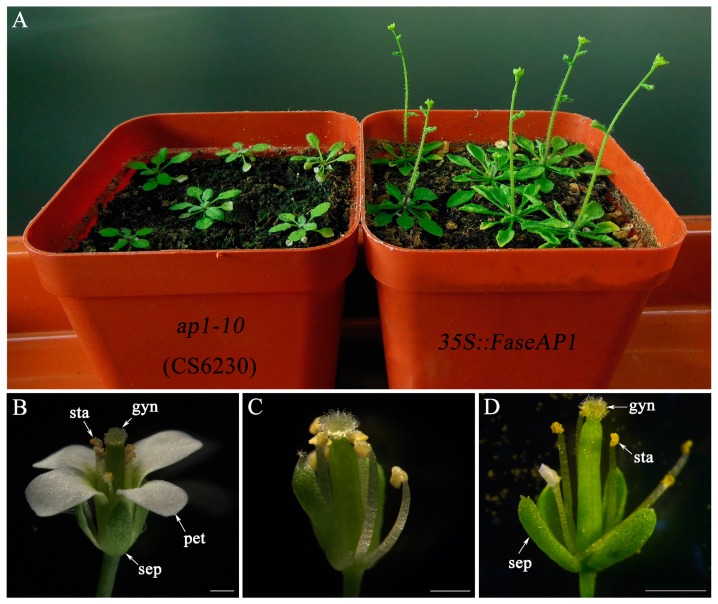
Phenotypes comparison of the wild-type, Arabidopsis *ap1-10* mutant and *35S::FaesAP1* transgenic Arabidopsis *ap1-10* mutant. (**A**) Arabidopsis *ap1-10* mutant and *35S::FaesAP1* transgenic Arabidopsis *ap1-10* mutant (*T-7/ap1-10*) cultured in the greenhouse under long-day condition (13 h light/11 h dark) at 25 °C light / 23 °C dark for 23 days; (**B**) wild-type Arabidopsis flower with normal 4 whorls of floral organs; (**C**) Arabidopsis *ap1-10* mutant flower with two carpelloid sepals in whorl 1 and the petal whorl missing. (**D**) T-15 independent line *35S:: FaesAP1* transgenic Arabidopsis *ap1-10* mutant flowers with four normal sepals in whorl 1, six stamens in whorls 2 but one filament attached with a petaloid structure, and a normal gynoecium in whorl 3. Sepal (sep); petal (pet); stamen (sta); gynoecium (gyn). Scale Bars: (**B**,**C**) 500 μm; (**D**) 1 mm.

Ten (66.67%) showed an obviously early flowering phenotypes and fast growth of vegetative organs, but with the rosette leaves and cauline leaves curling upward (Figure 8A), of which three lines produced flowers with petal whorl loss, and consisting of four normal sepals in whorl 1, six stamens in whorl 2 but one filament attached with a petaloid structure, and a normal silique-like gynoecium in whorl 3 (Figure 8D), and the remaining seven lines only displayed early flowering phenotypes. Moreover, five transgenic lines (33.33%) displayed no complementation. In addition, the phenotype changes of transgenic Arabidopsis corresponded to *FaesAP1* expression levels. For example, the *FaesAP1* expression in the transgenic lines showing strong complementation phenotypes were significantly higher than those lacking complementation (Figure 7) (*LSD, p* < 0.01). Furthermore, the *FaesAP1* expression in the transgenic lines only showing early flowering were significantly higher than those without complementation and lower than those with strong rescued phenotypes (Figure 7) (*LSD, p* < 0.05). 

## 3. Discussion

Usually, the undifferentiated petaloid perianth, defined as tepals, is commonly present in basal angiosperms, magnoliids and monocots, which does not completely correspond to the perianth differentiated into typical sepals and petals in core eudicots [16]. However, the perianth only comprising a single-whorl of five petaloid tepals is observed in *F. esculentum*, which make it an ideal model for exploring the perianth identity program in the early-diverging clades of core eudicots. In some core eudicots, *AP1* and its orthologs were proved to be involved in differential perianth (sepals and petals) development [1,7,8], while other *AP1* ortholog, such as *MPF3* from *Physalis floridana*, was only required for specifying calyx (sepals) identity [10]. In our study, *FaesAP1* expression was detected in all floral organs (tepals, stamens and gynoecia) of *F. esculentum*, and GUS reporter-gene driven by the *pFaesAP1* was expressed in all floral organs excluding petals and vegetable tissues in transgenic Arabidopsis. These results showed a broader expression zones than its orthologs in core eudicots [1,7,8], but a similar expression pattern with *AP1-/FUL*-like genes from eudicots or basal angiosperms [13,14]. Moreover, the *PISTILLATA* (*PI*) and *APETALA3* (*AP3*) orthologs commonly involving in specifying petal identity in most core eudicots are proved to be only responsible for stamen development in *F. esculentum* [29,30], which may suggest a different perianth identity program in *F. esculentum*. Furthermore, ectopic expression of *FaesAP1* in Arabidopsis *ap1-10* mutant fully complement sepal development in whorl 1 and produced flower with three whorls of floral organs (sepal whorl, stamen whorl and carpel whorl), which completely correspond to floral architecture of *F. esculentum* with one single whorl of undifferentiated tepals and inner two whorls of reproductive structure (stamen and carpel). We therefore provided evidence for the showy tepals in the *F. esculentum* is homologs to core eudicots sepals. In addition, these findings show a different perianth identity program in Caryophyllales and suggest that *AP1* orthologs involving in petal development may present independent evolution across different clades of core eudicots. Furthermore, ectopic expression of *FaesAP1* in Arabidopsis *ap1-10* mutant obviously prompt early flowering, which suggest that *FaesAP1* holds potential for biotechnical engineering to develop early flowering varieties of *F. esculentum*. 

## 4. Materials and Methods 

### 4.1. Plant Material

Pin and thrum floral buds at different developmental stages were sampled from *F. esculentum* ‘Beizaosheng’ growing under natural conditions in Jingzhou, China, respectively. The root, stem, cotyledon, juvenile leaves, inflorescence, perianth, stamen, gynoecium and young fruit were dissected, immediately frozen in liquid nitrogen, and then stored at −80 °C until used. The *Arabidopsis* Col-0 (Columbia ecotype) and *ap1-10* mutant line (CS6230, Landsberg ecotype) seeds were obtained from the ABRC (*Arabidopsis* Biological Resource Center, ABRC) at Ohio State University, USA.

### 4.2. Isolation and Characterization of FaesAP1 and FaesAP1 Promoter (pFaesAP1) from F. esculentum

Total RNA from various development stages floral buds and first-strand cDNA were prepared according to Li et al. [18]. The 3′ end and 5′ partial cDNA sequences of *FaesAP1* were obtained by using the 3′-full RACE Core Set Ver. 2.0 kit (TaKaRa, Shiga, Japan) with gene-specific primer GSPAP1 (5′- GTGATACCTGCATGGAGAAGATC -3′), and 5′ Full-RACE Kit (TaKaRa, Shiga, Japan) with gene-specific primers AP1GSP1 (5′- GATGGAAGTGGTGGGAAGAGTAGGA-3′) and AP1GSP2 (5′- CAGCTTTCCCCTATTGGAGAAAAC-3′) based on the manufacturer’s protocol, respectively. Full-length *FaesAP1* cDNA amplified followed by 3′- and 5′-RACE with the forward primer TFaesAP1F (5′- AGGATCCACAGAAGAGCAAAGAAGAAG-3′) and the reverse primer TFaesAP1R (5′- TCATACGTAGCAAGTCTGGTTTCACAC -3′) according to the protocol described by Li et al. [18]. Sequence alignments and phylogenetic analysis of *FaesAP1* were referenced the method described by Liu et al. [31]. Putative FaesAP1 protein sequences and various AP1/FUL-like proteins were selected for Phylogenetic trees from NCBI Genbank (Table S1). Moreover, the AGL6-like and SEP-like proteins were also included as outgroup because previous phylogenetic analysis grouping them into the AP1/SEP/AGL6 superclade [32,33].

Genomic DNA was extracted from *F. esculentum* juvenile leaves by using the CTAB Plant Genomic DNA Rapid Extraction Kit (Aidlab, Beijing, China) following the manufacturer’s protocol. The *FaesAP1* 5′ flanking region was isolated from buckwheat Genomic DNA by using Genome Walking Kit (TaKaRa, Japan) following the manufacturer’s protocol and with gene-specific primer D1AP1SP1 (5′-ACAATAAGGCCAACTTCAGCATCAC-3′), D1AP1SP2 (5′- AAAGTCACTTGCCTGTTGATCTTGT-3′), and D1AP1SP3 (5′- TTTGCTCTTCTGTCGCTCACTGCTT-3′) for the first walking sequencing, and with gene-specific primer D2AP1SP1 (5′- TGTTGTTTCTGGTGTAAGCAAGGAC-3′), D2AP1SP2(5′- TTGCTGAGAATGGACATCATAGAAT-3′), and D2AP1SP3(5′- TGTACGTAGAAGAAGATCGAGAACG-3′) for the second walking sequencing. The *FaesAP1* promoter was amplified by PCR with the forward primer pFaesAP1F (5′- GAGCTCTTGTTTCACTGCAATAGGTTCACCTG-3′) and the reverse primer pFaesAP1R (5′- TCTAGATTCTTCTCTTCTTCTTTGCTCTTCT -3′) base on two walking sequences. The putative transcription start site was found according to the methods described by Solovyev et al. [34], and the *cis-acting* Regulatory DNA elements of the *FaesAP1* promoter were found in the PLACE database [35].

### 4.3. Cytomorphological Examination and Expression Analysis of FaesAP1

Pin and thrum flowers buds of *F. esculentum* ‘Beizaosheng’ were sampled at sequential developmental stages, and subsequently fixed, dehydrated, cleared, infiltrated, embedded into paraffin block, serially sectioned, and then sections were stained and observed with photomicrographs taken according to Liu et al. [31].

*FaesAP1* expression was detected in pin and thrum flower by semi-quantitative RT-PCR (sqRT-PCR) and quantitative real-time PCR (qPCR) with the gene-specific primers QFaesAP1F (5′-CAACATGCTGGTCAACAAGATC-3′) and QFaesAP1R (5′-TATAGGCTCAAGGGTAAGCTC-3′). Amplification fragment of *F. esculentum actin* gene (HQ398855.1) with the forward primers QFaesactinF (5′-ACCTTGCTGGACGTGACCTTAC-3′) and the reverse QFaesactinR (5′-CCATCAGGAAGCTCATAGTTC-3′) was used as a positive control.

For sqRT-PCR analysis, 1 µg of total RNA was extracted from root, stem, cotyledon, juvenile leaves, inflorescence, tepal, stamen, gynoecium and young fruit of *F. esculentum* respectively. Total RNAs and first-strand cDNAs were prepared according to protocol described above but using an oligo (dT)18 primer. The PCR amplification was performed for 25 cycles as follows: 30 s at 94 °C, 30 s at 58 °C and 30 s at 72 °C, preceded by 3 min at 94 °C and followed by 5 min at 72 °C. 20 µl of the total PCR product (50 µL) in each reaction by electrophoresis in a 1% agarose gel, and then taken photography using GelDoc^TM^ XR+ Gel Imaging Systems (Bio-Rad, Hercules, CA, USA). 

For qPCR analysis, total RNAs from pin/thrum floral buds at various developmental stages were extracted with the procedure described above. DNA free total RNA and first-strand cDNA were prepared by using the HiScript^®^ II Q RT SuperMix for qPCR kit (Vazyme, Nanjing, China) following the manufacturer’s protocol. Quantitative real-time PCR (qPCR) with three biological replicates was carried out using Line-Gene 9600 Plus Real-time PCR Detection System, with SYBR green I for transcript measurements. The reaction mixture was cycled as follows: 95 °C for 30 s, followed by 40 cycles of 95 °C for 10 s, 60 °C for 30 s, and then followed by melt curve stage for 95 °C for 15 s, 60 °C for 60 s, and 95 °C for 15 s. The experiments were repeated three times for each sample. 

### 4.4. Construction of Promoter-GUS Fusions, Arabidopsis Transformation and Histochemical GUS Assay

The 2.2 kb 5′ flanking region upstream of *FaesAP1* translation start was cloned into pCAMBIA1300 vector with *Xba* I and *Sac* I restriction enzymes. *pFaesAP1*::*GUS* construct was transformed into *A. thaliana* Col-0 plants (ecotype Columbia) using the floral-dip method described by Clough and Bent (1998) [36]. Transgenic Arabidopsis seeds were selected, and seedlings were cultivated according to Liu et al. [37]. The T_1_ daughter lines of independent transgenic plants were prepared for histochemical GUS staining.

Different tissues of transgenic Arabidopsis were fixed in 90% acetone for 20 min at 4 °C and then discarded the liquids, Washed the tissues with GUS assay buffer containing 50 mM sodium phosphate (pH 7.0), 1 mM K_3_Fe(CN)_6_, 1 mM K_4_Fe(CN)_6_·3H2O, 10 mM EDTA(pH 8.0), 0.2 % and Triton X-100(*v/v*) two times, followed by incubated in a mixture of GUS assay buffer and 2 mM X-Gluc for 12 h at 37 °C, removed the liquids and later cleared in a graded ethanol series (75, 85, 95 and 100%). The samples were observed under a Leica 165C microscope, and photomicrographs were taken.

### 4.5. Ectopic Expression Analysis of FaesAP1 in Arabidopsis ap1-10 Mutant

Full-length *FaesAP1* cDNA in the sense orientation were cloned into pBI121 vector (BD Biosciences, Clontech) with *Xba* I and *Sac* I restriction enzymes under control of the CaMV35S promoter. The *35S::FaesAP1* construct were transformed into *Arabidopsis ap1-10* mutant using the floral-dip method described above. Transgenic Arabidopsis seeds were selected, and seedlings were cultivated according to Liu et al. The phenotypes of transgenic *Arabidopsis ap1-10* mutant lines were analyzed. The complementation degrees of independent transgenic lines of *35S::FaesAP1 Arabidopsis ap1-10* mutant were categorized as ‘no complementation’, ‘medium complementation’ and ‘strong complementation’. Moreover, two independent transgenic lines of each complementation degree were confirmed by qPCR with the primers QFaesAP1F and QFaesAP1R suggested above, respectively. Amplification fragment of *A. thaliana β*-*actin* with the primers qactinF (5′-GATTTGGCATCACACTTTCTACAATG-3′) and qactinR (5′-GTTCCACCACTGAGCACAATG-3′) was as a positive control. 

### 4.6. Statistical Treatment

All experiments were carried out with three biological replicates, and data were expressed as mean ± SD (standard deviation). Statistical significance was determined by *LSD*, and statistical significance was declared at *p*-value ≤ 0.01 or 0.05, respectively.

## Supplementary Materials

Supplementary materials can be found at https://www.mdpi.com/1422-0067/20/8/2021/s1.

## Abbreviations

PCR	Polymerase Chain Reaction
qPCR	Quantitative real-time PCR
sqRT-PCR	Semi-quantitative RT-PCR
LSD	Least Significant Difference
