# Supplementary material for: Ectopic Expression of a Fagopyrum esculentum APETALA1 Ortholog only Rescues Sepal Development in Arabidopsis ap1 Mutant
Source: Int J Mol Sci. 2019 Apr 24;20(8):2021. doi: 10.3390/ijms20082021 (PMC6515404; doi:10.3390/ijms20082021)
Supplement: Supplementary file 1 [file ijms-20-02021-s001.pdf]

***> pFaesAP1***

−1959 TTGTTTCACT G **CAAT**AGGTT CACCTGTTCC A **AGAAA**AACA AAACAAAACA AAACGAAACT

CAAT-box POLLEN1LELAT52

−1899 TGTATAATCT CCAAGTAAG A TGCTTGCTGA AATATTC AT**C ACATG** TAATT TTG **CATCTG** C

MYCCONSENSUSAT

−1839 ATATAGCAAG TTTTTTCTG T AAGGATTAGG TTGA **TACT**TG TATTCATCAT GATAGA **TACT**

CACTFTPPCA1 CACTFTPPCA1

−1779 TAGTACC**CAT TTG** GTTGGAA CAGTGGAATG G AATAAAATA AGGGCCCGTT TGGTTTTAAC

MYCCONSENSUSAT

−1719 TCCGGAATCG GAAACGAAT C TAGTCCATAC GTATAGTTTT ATTCATGAAA **GTTTTTG** AAA

SEF4MOTIFGM7S

−1659 TCATTCCTTG TGCTTTGAA G TAATTGGAGT AGAGTTA AAT **TACT**TTTGAT ACTCTATAGG

CACTFTPPCA1

−1599 TAACGAAATG TGGGGAAAC A AGTGCATTTT GCATT **AAACC A** AAATGAATG CTTCCATAAA

MYB1AT

−1539 ATT**TCACGA** A TAA **GGATA**CG TATATGGACT AAATTC GTTT CCATTCCATT TCCG TTTTCG

RHERPATEXPA7 MYBST1

−1479 TAGTTGGAAC TAAATGGGCC CTAAAATTGA AT**AAACCA**TA A**AGAAA**GGAA ATGGAATTGA

MYB1AT POLLEN1LELAT52

−1419 TGAGA **TTATT T**TCTAGTATA AAATGACCTC T**AACCAAAC** A TA**GTTTTTG** A TTAATTGGAA

TATA-box MYBPLANT SEF4MOTIFGM7S

−1359 TGATTCCTAG CGGTTCCAA A CGAACCCTAA ATTTATG AAA CTTC **TAACTG** ATGAATTTGC

MYB2AT

−1299 AGGAAGCCTA CATATC**CAAT** TTGAAGAACA AATCAAAC CT TTAGTATCCT GAACC **ACAAA**

CAAT-box XYLAT

−1239 **GAA**CCATTGA TGCTGCTATA TTTGATCATC AAAC**AGAAA**G AACTTGTCAG ATTGAAG GCT

POLLEN1LELAT52

−1179 CATTTCGAAC TATATACGTG CATTAAGCTG TAAGCCC AAC AATCTGAACT TTTTC CATGA

−1119 CTGCATACAA GCTAAGTAAG GATTTGT**CAT CTG**GGTTAGG GTAAAAAAAA C TCC**AGAAA**A

MYCCONSENSUSAT

−1059 TGAAATTGGG GCGAATCTTT TGAGATTG**CA AT**ATTGAAAC CCT **AGAAA**GA ATGGAAACAA

CAAT-box POLLEN1LELAT52

−999 AGGCATGGAA TATGATAGTG CTGAAGTGAA AAAGCC ATGG GAATCTGAAT GAGATGTCAA

−939 GTGCAC**ACAA AGAA**A**AGAAA** CAAAAGTAAA AAAGTAGAAT GAGGTGTTGT TCCACGGAAG

XYLAT POLLEN1LELAT52

−879 CTAGTTGGAA AACTAATTAC T**CCGAC**TCTA TGATTTTGAC ATGG GTGGGG TTC **CAACTG**T

LTRECOREATCOR15 MYCCONSENSUSAT

−819 CGGCACTCAA ACTATTCAA A TCTT **CAAT**CA AGAGTTCTTC TACTAG GGTT TAAGTAATTT

CAAT-box

−759 TGT**CAAT**TAA GATAAACCTA CTTTT CTACA TAATCCCATA TCA GTAGGGC GCCGTCAGCA

CAAT-box

−699 CAAGGATGAC GTCCGTACAA CCCAAATAAG TGTAGACGAA CCAGAAC ACG ACAGCACACC

**Figure S1:** FaesAP1 promoter sequence. The transcription start site (+1) is in bold and boxed. The start codon ATG is in bold and boxed. Putative cis-acting regulatory elements are in bold and underline.

**Table S1:** Information on Sequences selected for alignments and phylogenetic analyses from NCBI GenBank.

| Taxon and species | Protein name | Accession numbers | lineage |
|---|---|---|---|
| Brassicaceae | | | |
| *Arabidopsis thaliana* | AP1 | P35631.2 | AP1/FUL |
| | euFUL | Q38876.1 | AP1/FUL |
| | AGL79 | AAN52802.1 | AP1/FUL |
| | SEP1 | P29382.2 | SEP |
| | SEP2 | P29384.1 | SEP |
| | SEP3 | NP_850953.1 | SEP |
| | SEP4 | P29383.2 | SEP |
| | AGL6 | AEC10582.1 | AGL6 |
| *Cardamine hirsuta* | CahiAP1 | AQQ16907.1 | AP1/FUL |
| Polygonaceae | | | |
| *Fagopyrum esculentum* | FaesAP1 | AKI81897.1 | AP1/FUL |
| Poaceae | | | |
| *Oryza sativa* | OsMADS14 | Q10CQ1.2 | AP1/FUL |
| | OsMADS15 | Q6Q9I2.2 | AP1/FUL |
| | OsMADS18 | Q0D4T4.1 | AP1/FUL |
| | OsMADS20 | Q2QQA3.2 | AP1/FUL |
| | OsMADS5 | Q0DEB8.1 | SEP |
| | OsMADS7 | Q0J466.2 | SEP |
| | OsMADS8 | Q9SAR1.1 | SEP |
| | OsMADS34 | Q6Q9H6.2 | SEP |
| | OsMADS6 | Q6EU39.1 | AGL6 |
| Orchidaceae | | | |
| *Dendrobium* hybrid cultivar | DOAP1 | ARI44760.1 | AP1/FUL |
| *Oncidium* hybrid cultivar | OAP1 | ADJ67240.1 | AP1/FUL |
| | OSEP3 | ADJ67238.1 | SEP |
| | OSEP1/2 | ADJ67241.1 | SEP |
| | OAGL6 | ADJ67239.1 | AGL6 |
| Liliaceae | | | |
| *Lilium longiflorum* | LMADS5 | ADT78582.1 | AP1/FUL |
| | LMADS6 | ADT78583.1 | AP1/FUL |
| | LMADS7 | ADT78584.1 | AP1/FUL |
| Chloranthaceae | | | |
| *Chloranthus spicatus* | CsAP1 | AAQ83693.1 | AP1/FUL |
| Ranunculaceae | | | |
| *Nigella damascena* | NdFL1 | ALM95510.1 | AP1/FUL |
| | NdFL2 | ALM95511.1 | AP1/FUL |
| | NdSEP3 | ALM95519.1 | SEP |
| | NdSEP2 | ALM95518.1 | SEP |
| | NdSEP1 | ALM95517.1 | SEP |
| | NdAGL6 | ALM95509.1 | AGL6 |
| *Aquilegia coerulea* | AqFL1a | AGX01552.1 | AP1/FUL |
| | AqFL1b | AGX01553.1 | AP1/FUL |
| Berberidaceae | | | |
| *Epimedium sagittatum* | EsFUL | AEX58637.1 | AP1/FUL |
| | EsAGL2-1 | AEX58639.1 | SEP |
| | EsAGL2-2 | AEX58640.1 | SEP |
| | EsAGL6 | AEX58638.1 | AGL6 |
| Eupteleaceae | | | |
| *Euptelea pleiosperma* | EuplFL1 | ABG49518.1 | AP1/FUL |
| | EuplFL2 | ABG49519.1 | AP1/FUL |
| | EuplSEP1 | ADC79707.1 | SEP |
| | EuplSEP3 | ADC79706.1 | SEP |
| Cercidiphyllaceae | | | |
| *Cercidiphyllum japonicum* | CejaFUL | ASY97766.1 | AP1/FUL |
| | CejaAP1 | ASY97763.1 | AP1/FUL |
| | CejaAGL6 | ASY97761.1 | AGL6 |
| Vitaceae | | | |
| *Vitis vinifera* | VAP1 | AAT07447.1 | AP1/FUL |
| | VFUL | AAT07448.1 | AP1/FUL |
| Rosaceae | | | |
| *Prunus persica* | PpMADS1 | ABU63953.1 | AP1/FUL |

| | | | |
|---|---|---|---|
| | PperFUL | CAJ28929.1 | AP1/FUL |
| *Kerria japonica* | KejaAP1 | AXR86366.1 | AP1/FUL |
| | KejaSEP1 | AXR86364.1 | SEP |
| | KejaSEP3 | AXR86365.1 | SEP |
| Passifloraceae | | | |
| *Passiflora edulis* | PaedAP1 | AQN67666.1 | AP1/FUL |
| | PaedFUL | AQN67667.1 | AP1/FUL |
| | PaedSEP1 | AET98846.1 | SEP |
| Euphorbiaceae | | | |
| *Jatropha curcas* | JcAP1 | AKM06060.1 | AP1/FUL |
| *Plukenetia volubilis* | PlvoAP1 | ANA05340.1 | AP1/FUL |
| Salicaceae | | | |
| *Salix discolor* | SAP1 | AAY82245.1 | AP1/FUL |
| Chenopodiaceae | | | |
| *Spinacia oleracea* | SpAP1-1 | ACE75943.2 | AP1/FUL |
| | SpAP1-2 | ACE75944.2 | AP1/FUL |
| | SpFUL | ACE75945.2 | AP1/FUL |
| Theaceae | | | |
| *Camellia japonica* | CjAPL1 | AFW15783.1 | AP1/FUL |
| | CjAPL2 | AFX74875.1 | AP1/FUL |
| | CjAGL6 | AFW15784.1 | SEP |
| Rubiaceae | | | |
| Coffea arabica | CaAP1 | AHW58038.1 | AP1/FUL |
| | CaFUL | AHW58040.1 | AP1/FUL |
| | CaAGL6 | AHW58046.1 | AGL6 |
| | CaSEP1/2 | AHW58036.1 | SEP |
| | CaSEP3 | AHW58034.1 | SEP |
| | CaSEP4 | AHW58033.1 | SEP |
| Solanaceae | | | |
| *Solanum lycopersicum* | MADS-MC | NP_001234665.1 | AP1/FUL |
| | TDR4 | CAA43169.1 | AP1/FUL |
| | MBP7 | NP_001294867.1 | AP1/FUL |
| | SlAGL6 | NP_001348459.1 | AGL6 |
| Scrophulariaceae | | | |
| *Antirrhinum majus* | SQUA | CAA45228.1 | AP1/FUL |
| | DEFH28 | AAK72467.1 | AP1/FUL |
| Asteraceae | | | |
| *Gerbera* hybrid cultivar | GSQUA1 | CAA08805.2 | AP1/FUL |
| | GSQUA3 | CAX65662.1 | AP1/FUL |
| | GSQUA2 | CAX65661.1 | AP1/FUL |
| | GSQUA5 | CAX65663.1 | AP1/FUL |
| | GSQUA6 | CAX65664.1 | AP1/FUL |
| | GRCD6 | ASP44958.1 | SEP |
| | GRCD7 | ASP44959.1 | SEP |
| | GRCD8 | ASP44960.1 | SEP |
| *Chrysanthemum* x *morifolium* | CDM8 | AAO22981.1 | AP1/FUL |
| | CDM41 | AAO22980.1 | AP1/FUL |
| | CDM111 | AAO22979.1 | AP1/FUL |
